# COPD-Lower Respiratory Tract Infection Visual Analogue Score (c-LRTI-VAS) validation in stable and exacerbated patients with COPD

**DOI:** 10.1136/bmjresp-2020-000761

**Published:** 2021-02-16

**Authors:** Hendrik Johannes Prins, Ruud Duijkers, Johannes M A Daniels, Thys van der Molen, Tjip S van der Werf, Wim Boersma

**Affiliations:** 1 Department Pulmonary Diseases, Noordwest Ziekenhuisgroep, Alkmaar, Noord-Holland, The Netherlands; 2 Pulmonary Diseases, Vrije Universiteit Amsterdam, Amsterdam, Noord-Holland, The Netherlands; 3 Department of Pulmonary Diseases & Tuberculosis, University Medical Centre Groningen, Groningen, Groningen, The Netherlands; 4 Infectious diseases Service and Tuberculosis unit, University of Groningen Faculty of Medical Sciences, Groningen, Groningen, The Netherlands; 5 pulmonary disease, Noordwest Ziekenhuisgroep, Alkmaar, Noord-Holland, The Netherlands

**Keywords:** COPD exacerbations

## Abstract

**Background:**

We developed the chronic obstructive pulmonary disease (COPD)-Lower Respiratory Tract Infection-Visual Analogue Score (c-LRTI-VAS) in order to easily quantify symptoms during exacerbations in patients with COPD. This study aimed to validate this score.

**Methods:**

In our study, patients with stable COPD as well as those with an acute exacerbations of COPD (AECOPD) were included. The results of c-LRTI-VAS were compared with other markers of disease activity (lung function parameters, oxygen saturation and two health related quality of life questionnaires (St Georges Respiratory Questionnaire (SGRQ) and Clinical COPD Questionnaire (CCQ)) and validity, reliability and responsiveness were assessed.

**Results:**

Eighty-eight patients with clinically stable COPD and 102 patients who had an AECOPD completed the c-LRTI-VAS questionnaire. When testing on two separate occasions for repeatability, no statistically significant difference between total scores was found 0.143 (SD 5.42) (p=0.826). Internal consistency was high across items (Cronbach’s apha 0.755). Correlation with SGRQ and CCQ total scores was moderate to high. After treatment for hospitalised AECOPD, the mean c-LRTI-VAS total score improved 8.14 points (SD 9.13; p≤0.001).

**Conclusions:**

c-LRTI-VAS showed proper validity, responsiveness to change and moderate to high correlation with other questionnaires. It, therefore, appears a reliable tool for symptom measurement during AECOPD.

**Trial registration number:**

NCT01232140.

**Key messages**

- Is the chronic obstructive pulmonary disease (COPD)-Lower Respiratory Tract Infection Visual Analogue Score (LRTI-VAS) a valid instrument in stable and exacerbated COPD?
- The LRTI-VAS showed proper repeatability and responsiveness, moderate to high correlation with other validated questionnaires and a moderate internal consistency.
- This validated questionnaire can be used to quantify COPD symptoms in an easy and non-invasive way instead of measuring the impact of symptoms on daily life and well-being.

## Background

Chronic obstructive pulmonary disease (COPD) is the fourth-leading cause of mortality worldwide and an important cause of morbidity.^1^ COPD is a common preventable and treatable disease that is characterised by persistent respiratory symptoms and airflow limitation and chronic low-grade local and systemic inflammation that is due to airway and/or alveolar abnormalities usually caused by significant exposure to noxious particles or gases.^1 2^ Clinical measures such as forced expiratory volume 1 s (FEV_1_) or oxygen saturation correlate only moderately with functional capacity of patients with COPD.^3 4^ The main determinants of a patient’s health-related quality of life (HRQL) appear to be the degree of dyspnoea, fatigue, muscle wasting, sleep and mood disturbances.^5 6^ Measurement of these symptoms and signs is very useful in monitoring patients with COPD. It is a strong predictor of future disease outcome and potentially modifies treatment management.^7^ To this end, many questionnaires have been developed to measure the impact of symptoms on quality of life.^8 9^ Although several questionnaires now exist that measure symptoms in a subdomain, validated specific questionnaires that solely focus on symptoms are in short supply.^10^ The one questionnaire available is comprehensive, but unfortunately, time consuming, and less suitable for bedside usage. An alternative for this problem would be a Visual Analogue Score (VAS). VAS has been used in many settings since their first description for the measurement of pain in 1957. VAS is known to be used at the bedside.^11^ Additionally, VAS can also be used for the quantification of respiratory symptoms such as dyspnoea, cough and sputum volume in COPD.^12^ To date, no questionnaire measuring symptoms has been properly validated in acute exacerbations of COPD (AECOPD). The incentive for the development of a practical health status instrument, the COPD-Lower Respiratory Tract Infection-VAS (c-LRTI-VAS) arose from routine clinical management of COPD. In daily practice, clinicians require a simple questionnaire designed to provide practitioners with standardised, reliable and valid information for assessing symptoms in AECOPD. This can be used for quantify duration of AECOPD, exacerbation severity and pattern of recovery. Which is particular important in trials studying treatment effect of AECOPD. The LRTI-VAS was used before to quantify symptoms in AECOPD but was not validated before.^13–15^ However, it was recently validated in non-CF bronchiectasis and since then adopted by the European Bronchiectasis Registry.^16^ On all occasions, the LRTI-VAS was generally well accepted by patients, and showed a high response rate. The aim of this study was to validate the c-LRTI-VAS for assessment of symptoms in patients with COPD in stable condition and during an AECOPD.

## Material and methods

### Study population

From November 2011 to November 2014, clinically stable patients with COPD visiting the outpatient clinic of the Department of Pulmonary Medicine of the Medical Centre Alkmaar, a large teaching hospital, were asked to participate by the primary investigator.^16^ A stable situation was defined as not having had an AECOPD defined by Global Initiative for Chronic Obstructive Lung Disease (GOLD) <1 month before study entry, no recent change in COPD associated medication <1 month before study entry. Also, immunocompromised patients or patients with respiratory disease other than COPD were excluded from participation. Data from patients with an AECOPD were available from a randomised clinical trial performed between July 2011 and February 2015. The study population consisted of patients diagnosed with COPD stages I–IV as defined by the GOLD, and a minimum smoking history of 10 pack years.^1^ All patients provided written informed consent in both patients groups. All patients provided their written informed consent.

### Development of the c-LRTI-VAS

**Figure 1:**
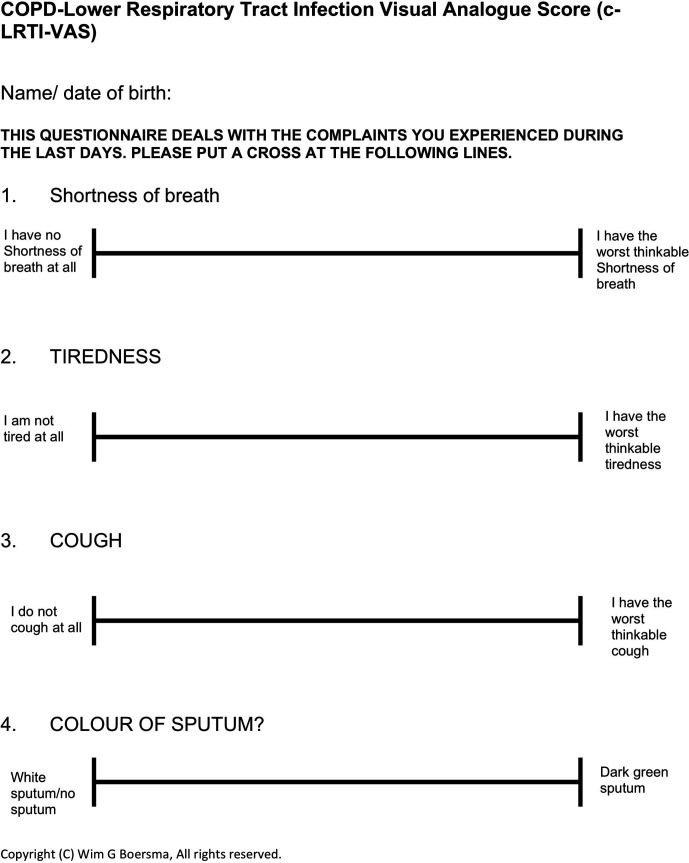
c-LRTI-VAS.

The initial specifications for the c-LRTI-VAS identified that the questionnaire should only contain the symptoms that physicians consider to be the most important for estimating the clinical status of the airways. Therefore, item generation was performed based on Anthonisen criteria with the addition of the symptom: fatigue.^17^ Fatigue was added as being one of the most prominent symptoms in COPD.^18^ A VAS scale was chosen to meet the specification of simplicity. The c-LRTI-VAS is short (four items) and easy to complete (figure 1). It takes patients approximately 1 min to complete the questionnaire, and assistance is generally not required. Patients were instructed to recall their experiences during the last day. They respond to each question using a VAS scale. The scale ranges from 1 to 10, the subjects being unaware of the numbers. Higher scores indicate more severe symptoms. Four symptom domains are scored: shortness of breath, tiredness, cough and sputum colour. Separate scores are calculated for each symptom and a total score is provided, consisting of the addition of all symptom scores. Similar weight is assigned to all symptom domains. For the present study, a Dutch version was used.

### Patient involvement

Patients with COPD and their families were not involved in setting the research questions or the outcome measures.

### Other questionnaires

Clinical COPD Questionnaire (CCQ) is defined as a disease-specific questionnaire that consists of 10 items. The items are divided into three different domains (functional state, symptoms and mental state) which can be scored separately. Added together they provide a total score, representing the impairment of quality of life. The CCQ requires about 4 min to complete^8 19^

St George Respiratory Questionnaire (SGRQ) is defined as a condition specific HRQL measure that consists of 76 items. These items are partitioned into three sections (symptoms, activity, impact), which are scored separately and can be added together to provide a total score, ranging from 0% to 100%. Zero indicates no impairment of quality of life. The SGRQ requires about 10 min to complete.^9^

### Study visits

All participants with stable COPD visited our outpatient clinic on two separate occasions 30 days apart. On both occasions, participants were asked to complete the LRTI-VAS, the CCQ and SGRQ. In addition, spirometry was measured. In case of participants with AECOPD, the first study visit was scheduled within 24 hours after the admission for AECOPD and 30 days after patients visited our outpatient clinic. On both occasions, patients were asked to complete the c-LRTI-VAS, CCQ and SGRQ. In addition, arterial oxygen saturation was measured using a fingertip pulse oximeter (Beurer Y23/003700, Ulm, Germany).

### Sputum colour analysis

Sputum samples were collected on the first day after admission and 1 month after admission. At the laboratory for microbiology, sputum colour was assessed with a previously validated five-point sputum colour chart (BronkoTest; Heredilab, Salt Lake City, Utah, USA) by specifically instructed analysts.^20^ This data were used to assess the correlation between reported sputum colour compared with objectified sputum colour.

### Validity of the c-LRTI-VAS

Patients with clinical stable COPD and patients with an AECOPD completed the c-LRTI-VAS, the CCQ and the SGRQ on two separate occasions. In addition, in patients with stable COPD spirometry was performed as well as pulse oxygen saturation measurement on both occasions. Correlation of c-LRTI-VAS, CCQ and SGRQ, FEV_1_, forced vital capacity (FVC) and oxygen saturation was calculated in order to test validity. Internal consistency was calculated in order to test the degree of association between the questionnaire items. In the group of patients with stable COPD, the c-LRTI-VAS, CCQ and SGRQ were administered and readministered after 1 month. On both occasions, patients conducted spirometry and pulse oxygen saturation measurement. These data were used to assess retest reliability. Patients were excluded if they had an exacerbation, an increase of respiratory symptoms due to heart failure or upper respiratory infection or a change in smoking status. An exacerbation was defined as an acute event characterised by worsening of the patient’s respiratory symptoms that is beyond normal day-to-day variations and one that leads to a change in medication.^1^

### Responsiveness

Patients with an AECOPD completed the c-LRTI-VAS, the CCQ and the SGRQ on the first day of their exacerbation. The questionnaires were readministered after 30 days. The responsiveness of the c-LRTI-VAS was assessed by comparing changes in score in the CCQ and c-LRTI-VAS.

### Statistical analysis

Our sample size regarding patients with an AECOPD was based on the data of Daniels *et al*.^13^ An effect size of 5 was expected with an SD of 12. With alpha being 0.05 and beta being 0.20 a sample size of 92 patients on each measuring moment was needed. With our sample size regarding patients with stable COPD, we assumed a moderate correlation (0.3) between the scores on *t*=1 and *t*=2, with alpha being 0.05 and beta being 0.20 in a sample size of 85 patients. In the group of patients with AECOPD, a higher number of drop-out was anticipated. Therefore, we decided to include an additional 10 patients. In the group of patients with stable COPD, this was not anticipated and therefore only an additional three patients were added.

Data analysis was performed using SPSS V.20.0. Data are expressed as means (SD) unless stated otherwise. Paired t-test was used to compare LRTI-VAS domain and total scores on two occasions during clinical stability and at the start and end of an exacerbation. To evaluate normal distribution, Kolmogorov-Smirnov test was used. In case of skewed distribution, Wilcoxon’s signed ranks test was used. Pearson’s correlation and the intraclass correlation coefficient (ICC) was used to assess validity. Internal consistency of the LRTI-VAS was measured by applying Cronbach’s alpha to each of the component scores at entry; accepting >0.7 as sufficient. Nominal and ordinal variables were expressed using frequency tables, modus and median. Interval/ratio variables were expressed in terms of mean, SD and CIs. Bland and Altman graphs were made to assess the agreement between day 1 and day 30. When comparing two variables, a p value of <0.05 was considered as statistically significant.

## Results

**Figure 2:**
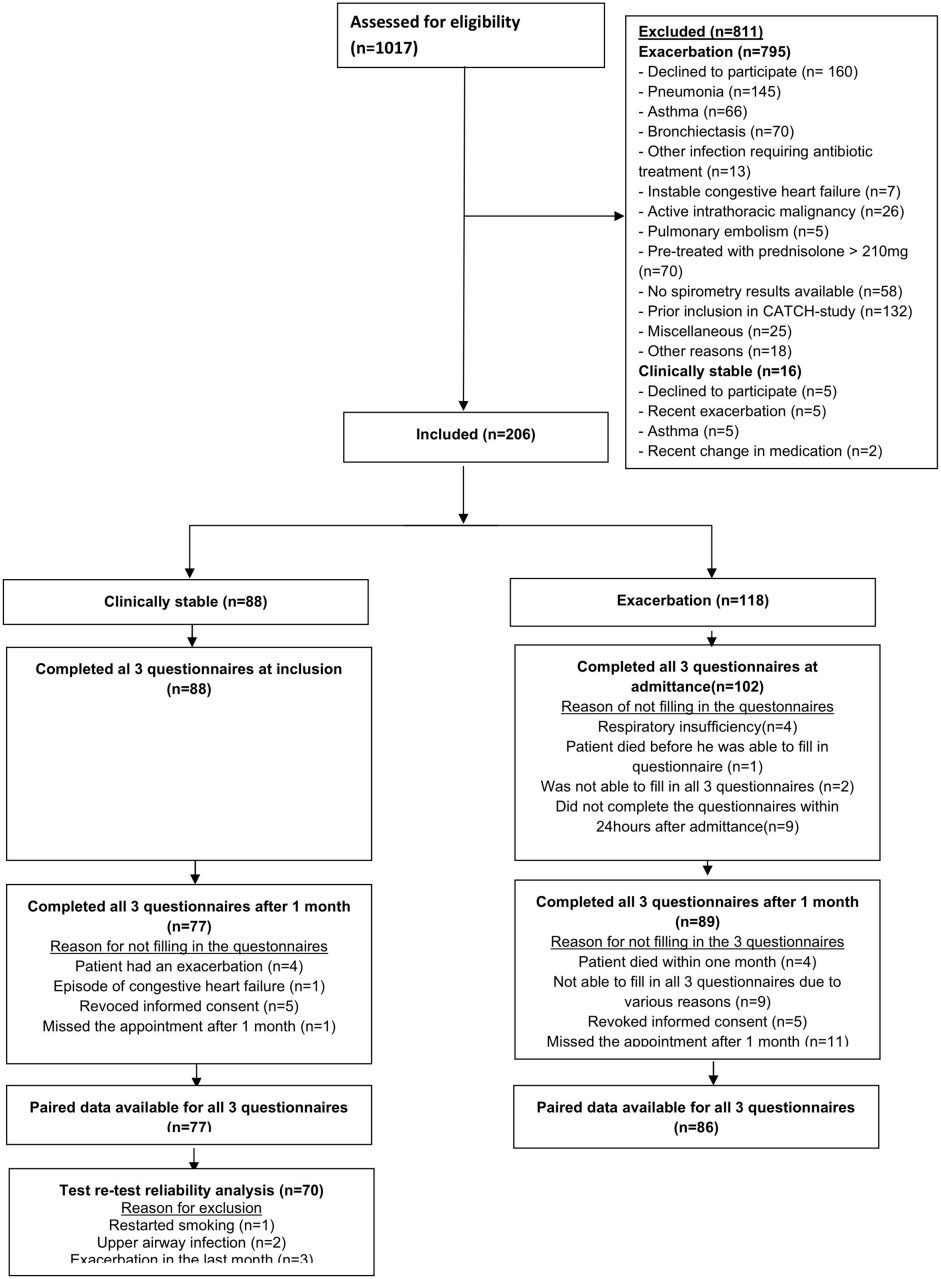
Trial profile.

Two hundred and six patients were included; 88 of whom were clinically stable and 102 that had an exacerbation (figure 2). Patients characteristics are shown in table 1.

**Table 1:** Baseline characteristics

	AECOPD (n=102)	Clinically stable (n=88)
Gender male (%)	44 (43.1)	56 (63.6)
Age, years	68.8 (10.4)	69 (12.5)
Current smoking n (%)	32 (31.4)	20 (23.5)
Pack-years	38.3 (18.4)	37.8 (15.1)
FEV1 % pred	46.8 (16.9)	54.2 (16.6)
FVC % pred	84.1 (21.7)	91.5 (16.5)
FEV1/FVC %	41.1 (12.4)	44.3 (11.6)
GOLD classification		
Stage I n (%)	7 (6.9)	7 (8.0)
Stage II n (%)	35 (34.3)	42 (47.7)
Stage III n (%)	45 (44.1)	37 (42.0)
Stage IV n (%)	15 (14.7)	2 (2.3)
number of exacerbations last year median (IQR)	1 (1–2)	0 (0–1)

All data are represented as mean (SD) unless specified otherwise.

AECOPD, acute exacerbation of COPD; FEV1, forced expiratory volume 1 s; FVC, forced vital capacity; GOLD, Global Initiative for Chronic Obstructive Lung Disease.

Eighty-six patients in the exacerbation group and 77 in the stable group completed all 3 questionnaires on two occasions. Median c-LRTI-VAS score during stable state was 11 (IQR 7–16) and during AECOPD, the mean was 23.2 (SD 6.2). Median CCQ score during the stable state was 2.25 (IQR 1.50–2.75), and during AECOPD, 3.88 (IQR 3.00–4.50). Mean SGRQ score during stable state was 44.1 (SD 21.2), and during AECOPD it was 63.5 (SD17.1) (please find results for the c-LRTI-VAS, CCQ and SGRQ domain scores in the (online supplemental file).

10.1136/bmjresp-2020-000761.supp1 Supplementary data

### Test–retest reliability

**Figure 3:**
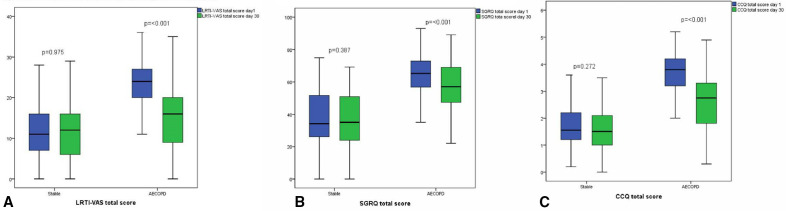
Scores of individual questionnaires during stable state and AECOPD at t=0 days and t=30 days. (A) c-LRTI-VAS, (B) SGRQ (C) CCQ. AECOPD, acute exacerbation of chronic obstructive pulmonary disease; CCQ, Clinical COPD Questionnaire; c-LRTI-VAS, COPD-lower respiratory tract infections-Visual Analogue Score; SGRQ, St George Respiratory Questionnaire.

**Figure 4:**
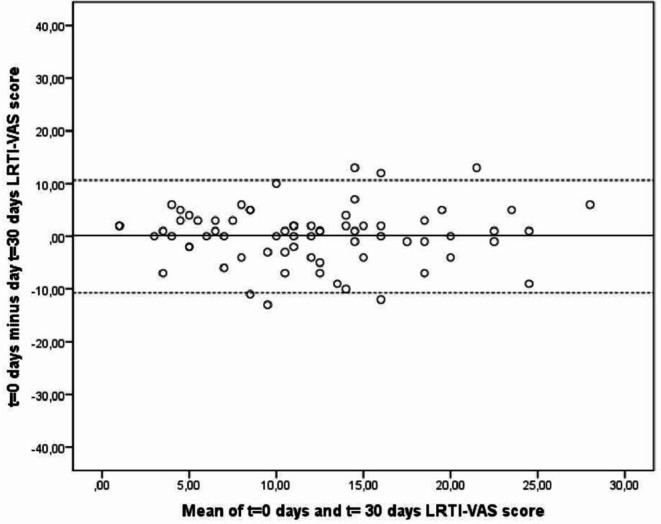
Bland-Altman plot c-LRTI-VAS. COPD-lower respiratory tract infections-Visual Analogue Score.

Seventy-seven patients with stable COPD completed all three questionnaires on day 1 and day 30. Six patients were excluded due to various reasons (figure 2). Mean difference of the c-LRTI-VAS was 0.143 (SD 5.42) (p=0.826) (figure 3). The ICC was 0.667 (95%CI 0.733 to 0.892, p<0.001) for the total c-LRTI-VAS score. The ICC of the SGRQ was 0.953 (95%CI 0.924 to 0.970, p<0.001). The ICC of the CCQ was 0.871 (95%CI 0.793 to 0.919, p<0.001). The relation between c-LRTI-VAS score on T=0 and T=30 is shown in the Bland and Altman plots (figure 4). No systematic errors can be seen as the mean difference was 0.143 with an upper limit of agreement of 10.775 and a lower limit of agreement of −10.480.

### Internal consistency

For the validation of internal consistency of the c-LRTI-VAS, both datasets of the AECOPD as well as the stable situation were merged (n=190). Cronbach’s alpha for the internal consistency for the 4 domains was 0.755, indicating a good consistency. Internal consistency increased when the item sputum purulence was deleted from the questionnaire to 0.803.

Cronbach’s alpha for the internal consistency during AECOPD (n=102) for the four domains was 0.533. Internal consistency increased further when sputum purulence was deleted to 0.642. Cronbach’s alpha for the internal consistency during the stable state (n=89) for the four domains was 0.623 Internal consistency increased further when the item sputum purulence was deleted from the questionnaire to 0.676. Internal consistency of SGRQ was 0.818 and of the CCQ 0.783

### Correlation

**Figure 5:**
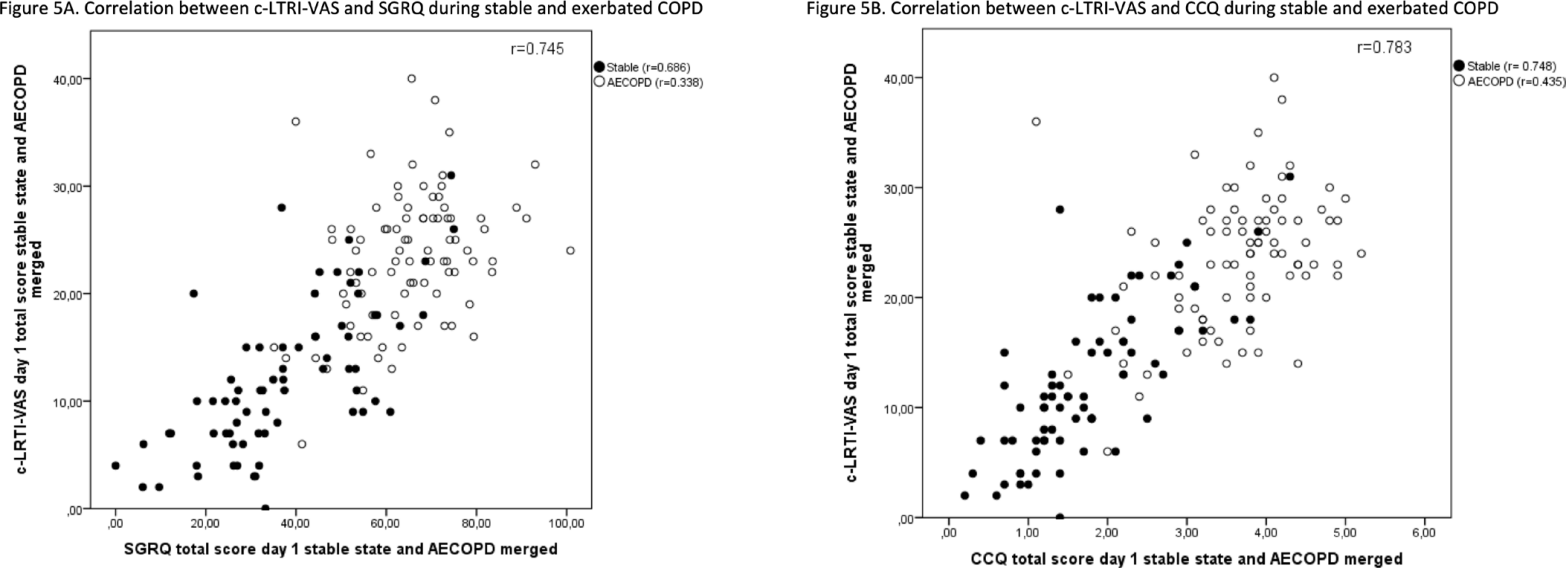
Correlation plot in stable state and during AECOPD. (A) c-LRTI-VAS and SGRQ (B) c-LRTI-VAS and CCQ. AECOPD, acute exacerbation of chronic obstructive pulmonary disease; CCQ, Clinical COPD Questionnaire; c-LRTI-VAS, COPD-lower respiratory tract infections-Visual Analogue Score; COPD, Chronic obstructive pulmonary disease; SGRQ, St George Respiratory Questionnaire.

The correlation coefficients between total scores on validated questionnaires (SGRQ and CCQ) are shown in figure 5. Correlation between FEV1, FVC, oxygen saturation, sputum colour and c-LRTI-VAS was low (r=0.071–0.377).

### Responsiveness

Eighty-six patients completed the c-LRTI-VAS, CCQ and SGRQ at admission and 1 month later. Mean difference of the c-LRTI-VAS was 8.14 SD 9.13 (95%CI 6.16 to 10.12 p=<0.001). Responsiveness for individual GOLD stages was shown in table 2.

**Table 2:** Responsiveness of c-LRTI-VAS according to GOLD stages

	c-LRTI-VAS T=0	c-LRTI-VAS t=30	Difference	P value
GOLD I (n=6)	22.5 (22.0–24.0)	9.5 (2.5–23.0)	−13.0 (-19.5;−1.0)	0.075
GOLD II (n=32)	23.5 (19.5–26.5)	16.0 (5.5–24.5)	−7.5 (-14.0;−2.0)	<0.001
GOLD III (n=35)	24.0 (19.0–27.0)	17.5 (7.0–26.0)	−6.5 (-12.0;−1.0)	0.001
GOLD IV (n=13)	24.0 (21.0–27.5)	16.0 (5.0–27.5)	−8.0 (-16;0.0)	0.013

All data are represented as median (IQR).

c-LRTI-VAS, COPD-lower respiratory tract infections-Visual Analogue Score; GOLD, Global initiative for chronic Obstructive Lung Disease.

## Discussion

This study shows that the c-LRTI-VAS questionnaire is valid, reliable and promises to be responsive to changes in patients with COPD. The VAS instrument has been around for a long time and initially mainly used for the quantification of pain. It has been shown to be reliable and is widely used. The VAS in COPD has mainly been used for quantification of dyspnoea, but has also been validated for the quantification of quality of life in COPD.^21–23^ Previously, we used the c-LRTI-VAS to quantify symptoms in 223 patients with AECOPD, and a slightly modified version of the LRTI-VAS was validated in a population of patients with bronchiectasis. During the validation study the LRTI-VAS showed moderate to high correlation with other validated questionnaires, responded to clinical changes and showed excellent repeatability and internal consistency.^13 16^ Currently many HRQL questionnaires are available such as the SGRQ, CCQ and COPD assessment test. All are comprehensive and do contain a domain of symptoms, but are not exclusively designed for measurements of symptoms.^8 9^ Although such an instrument was developed in the form of the EXACT-pro, this questionnaire still has the shortcoming that it is less suitable for illiterate or poorly educated patients compared with a VAS instrument.^10 24^ It was, therefore, thought that there is a need for a less extensive and time consuming questionnaire for patient-reported outcome in clinical settings that solely focusses on the most reported symptoms in COPD and that is suitable for poorly educated or illiterate patients. The items were generated based on the Anthonisen criteria and fatigue as being one of the most prominent features in COPD.^17 18^ Although other markers are able to monitor disease activity in AECOPD as pulmonary function tests, including peak flow or oxygen saturation, the c-LRTI-VAS is easy to administer and has a low burden on stable as well as on patients with an AECOPD. The VAS instrument has been used before in COPD, it has been used for the quantification of separate symptoms such as dyspnoea and cough as well as for the quantification of quality of life.^22 23 25^ Yet is has never been used solely for the quantification of the most frequent symptoms in stable COPD as well as in AECOPD.

In our population, subjects scored similar results on two separate occasions in a clinically stable situation. Patients during an outpatient visit scored within 1.2–4.1 points on the c-LRTI-VAS 10-point scale for shortness of breath, tiredness, cough and sputum purulence. During an exacerbation, scores for these symptoms increased to 5–8 points per item with a significant decrement 30 days after treatment for the exacerbation. Sputum purulence was only marginally increased during AECOPD compared with the recovered or stable state. This might be explained by the fact that patients’ assessment of sputum colour is unreliable as was shown earlier.^20^

COPD parameters such as lung function tests and oxygen saturation often do not correlate well with functional capacity and well-being.^3 4^ The absence of this relation may explain the low correlations we found between these parameters. This does not disqualify these parameters as they have important predictive values in COPD.^26 27^ Yet they do not play a significant role in quality of life as this is mainly defined by the presence and severity of symptoms such as dyspnoea, cough and fatigue.

The strength of our study is that patients with all GOLD-classes were included. An other strength is that the c-LRTI-VAS was validated for patients with stable COPD, as well as with AECOPD. Potential weaknesses were the high number of patients that were lost to follow-up. This potentially might have influenced our results. Another potential weakness is the generalisability of our results as this trial was performed in a hospital setting with patient admitted to hospital as well ambulant patients. It remains to be seen whether the LRTI-VAS is a useful tool in general practices.

The c-LRTI-VAS has shown to reliably measure shortness of breath, tiredness, cough and sputum colour, although sputum purulence proved not to contribute to the reliability and consistency of the questionnaire. This might be explained by the fact that not all patients routinely inspect their sputum, and so the answer given could be a ‘best guess’. Second, sputum colour can change rapidly, especially during acute exacerbations. And finally, sputum is not always homogeneous, which can be confusing. We, therefore, consider to adapt the c-LRTI-VAS by removing sputum purulence from the questionnaire. A potential replacement for sputum purulence might be anxiety as one of the most prominent features of patients with COPD.^5^ Yet we do think that the LRTI-VAS in its current form is a potentially valuable outcome measure when evaluating treatment effectiveness in clinical trials as it is easy to complete and to implement as was shown in earlier trials.

## Conclusion

The LRTI-VAS showed proper repeatability and responsiveness, moderate to high correlation with other validated questionnaires and a moderate internal consistency that was lowered by sputum purulence. The c-LRTI-VAS, therefore, meets all the criteria to be used in monitoring disease and can be used in clinical practice.
