# Supplementary material for: COPD-Lower Respiratory Tract Infection Visual Analogue Score (c-LRTI-VAS) validation in stable and exacerbated patients with COPD
Source: BMJ Open Respir Res. 2021 Feb 16;8(1):e000761. doi: 10.1136/bmjresp-2020-000761 (PMC7888334; doi:10.1136/bmjresp-2020-000761)
Supplement: Supplementary data [file bmjresp-2020-000761supp001.pdf]

Figure 1 supplementary information

COPD-Lower Respiratory Tract Infection Visual Analogue Score (c-LRTI-VAS)

Name/ date of birth:

THIS QUESTIONNAIRE DEALS WITH THE COMPLAINTS YOU EXPERIENCED DURING THE LAST DAYS. PLEASE PUT A CROSS AT THE FOLLOWING LINES.

1. Shortness of breath

I have no Shortness of breath at all	I have the worst thinkable Shortness of breath

2. TIREDNESS

I am not tired at all	I have the worst thinkable tiredness

3. COUGH

I do not cough at all	I have the worst thinkable cough

4. COLOUR OF SPUTUM?

White sputum/no sputum	Dark green sputum

Copyright (C) Wim G Boersma, All rights reserved.

Table 1 c-LRTI-VAS, CCQ and SGRQ score at inclusion in stable state and during AECOPD

c-LRTI-VAS stable t=0		c-LRTI-VAS AECOPD t=0	
Shortness of breath (median, IQR)	4(2-6)	Dyspnea (median, IQR)	7(6-9)
Tiredness (median, IQR)	3(1-6)	Tiredness (median, IQR)	8(5-9)
Cough (median, IQR)	2(1-4)	Cough (median, IQR)	6(5-8)
Sputum purulence (median, IQR)	1(0-2)	Sputum purulence (median, IQR)	2(0-5)
Total score (median, IQR)	11(7-16)	Total score (mean, SD)	23.2(6.2)
CCQ stable t=0		CCQ AECOPD t=0	
Symptoms (median, IQR)	2.25(1.50-2.75)	Symptoms (median, IQR)	3.88(3.00-4.50)
Mental (median, IQR)	0.50(0.00-1.50)	Mental (median, IQR)	2.50(1.38-3.50)
Functional (median, IQR)	1.50(0.75-2.50)	Functional (median, IQR)	4.00(3.19-4.75)
Total score (median, IQR)	1.70(1.20-2.30)	Total score (median, IQR)	3.70(3.18-4.1)
SGRQ stable t=0		SGRQ AECOPD t=0	
Symptoms (mean, SD)	44.1(21.2)	Symptoms (mean, SD)	63.5(17.1)
Activity (median, IQR)	66.1(47.5-73.8)	Activity (median, IQR)	86.3(73.1-92.5)
Impact (mean, SD)	26.6(17.7)	Impact (mean, SD)	55.4(18.0)
Total (mean, SD)	39.5(17.0)	Total (mean, SD)	64.9(13.8)
